# Comment on ‘Clinical Presentation and Early Outcomes of Congenital Endocrine Salt‐Wasting Syndromes Unrelated to 21‐Hydroxylase Deficiency’

**DOI:** 10.1002/edm2.70299

**Published:** 2026-07-28

**Authors:** Gulalai Tariq, Muhammad Faizan Khan, Malghalara

**Affiliations:** ^1^ Nowshera Medical College Nowshera Pakistan; ^2^ Khyber Medical University Peshawar Pakistan; ^3^ Jinnah Medical College Peshawar Pakistan

To the Editor,

1

We read with great interest the recent article by Ferrari et al. [1]. The authors should be congratulated for reporting one of the largest single‐centre cohorts of these exceptionally rare endocrine disorders and for providing valuable insights into their clinical presentation, diagnostic challenges and early outcomes. Given the rarity of these conditions, the study represents an important contribution to the paediatric endocrinology literature. However, we believe that several methodological aspects warrant further discussion because they may influence the interpretation of the study's principal conclusions regarding long‐term outcomes and disease evolution.

Firstly, we are concerned that the study's conclusions regarding preserved growth outcomes are based on a markedly reduced follow‐up cohort. Although 20 patients were initially enrolled, only 8 (40%) remained available for assessment at 36 months [1], with some diagnostic groups represented by only a single patient. Notably, the most treatment intensive group, systemic pseudohypoaldosteronism type 1 (PHA1s), described as having the greatest treatment burden, was reduced to a single patient at 36 months. Such selective loss to follow‐up of high burden cases introduces upward selection bias: the conclusion that growth outcomes were generally preserved may reflect only the subset of patients who tolerated the disease well enough to remain in care, systematically excluding more severely affected children who may have experienced growth impairment. This introduces bias away from the null, artificially inflating the apparent favourable prognosis. As highlighted by Howe et al., substantial loss to follow‐up in cohort studies can introduce selection bias and compromise the validity and generalizability of study findings [2].

Second, the cohort spans a 34‐year recruitment period (1989–2023), during which genetic testing evolved from clinical/biochemical diagnosis to molecular confirmation. The authors note that available molecular analyses were performed through targeted single‐gene testing, reflecting the diagnostic approach used at the time. This temporal evolution could partly confound the paper's key claim that disease‐specific features become more recognizable during follow‐up. This apparent emergence of disease‐specific features may reflect improved diagnostic capacity in 2023 versus clinical diagnosis in 1989, rather than true biological disease evolution. Consequently, longitudinal diagnostic clarification attributed to disease course may partly represent diagnostic advancement, biasing conclusions about natural disease trajectory away from the null. Numerous methodological studies and reporting guidelines have emphasized that observational studies should explicitly address potential sources of bias that may affect the interpretation of their findings, including temporal bias in long‐term retrospective cohorts [3].

Finally, the high early variability in mineralocorticoid dosing (CV 63%–69%) [1], may reflect differences in clinical management rather than true disease heterogeneity. Therefore, the reported clinical stabilization in aldosterone synthase deficiency may partly result from more standardized treatment over time rather than genuine disease evolution. Many studies have emphasized that clinical practice variation can influence observed treatment patterns [4].

The authors should be appreciated for conducting this valuable study and providing important insights into the long‐term outcomes of this rare condition. These considerations do not diminish the importance of this study. Rather, they highlight the need for cautious interpretation of long‐term outcome data derived from very small cohorts with substantial attrition and prolonged recruitment periods. Future multicenter prospective registries with standardized treatment protocols and complete longitudinal follow‐up will be essential to validate these important observations.

## Author Contributions

**Gulalai Tariq:** conceptualization, methodology, writing – original draft. **Muhammad Faizan Khan:** conceptualization, methodology, supervision, writing – review and editing. **Malghalara:** writing – original draft, methodology, investigation.

## Funding

The authors have nothing to report.

## Ethics Statement

The authors have nothing to report.

## Consent

The authors have nothing to report.

## Conflicts of Interest

The authors declare no conflicts of interest.

## Data Availability

Data sharing not applicable to this article as no datasets were generated or analysed during the current study.
